# Insult to Injury: Cross-Sectional Analysis of Preoperative Psychosocial Vulnerabilities in Adult Patients Undergoing Major Elective Cancer Surgery

**DOI:** 10.3390/cancers17172859

**Published:** 2025-08-30

**Authors:** Kurt S. Schultz, Samantha M. Linhares, Emily Y. Park, Elizabeth L. Godfrey, Uday Dhanda, Eliza J. Epstein, Kathryn Bailey Thomson Blake, Yuqing Huang, Haadia Zaheer, Ira L. Leeds

**Affiliations:** 1 Division of Colon & Rectal Surgery, Department of Surgery, Yale School of Medicine, New Haven, CT 06519, USA; kurt.schultz@yale.edu (K.S.S.); samantha.linhares@yale.edu (S.M.L.); emily.y.park@medstar.net (E.Y.P.); elizabeth.godfrey@yale.edu (E.L.G.); eliza.epstein@yale.edu (E.J.E.); baileythomson.blake@yale.edu (K.B.T.B.); yuqing.huang.yh745@yale.edu (Y.H.); 2 Investigative Medicine Ph.D. Program, Yale Graduate School of Arts and Sciences, New Haven, CT 06511, USA; 3 Department of Chronic Disease Epidemiology, Yale School of Public Health, New Haven, CT 06519, USA; uday.dhanda@yale.edu; 4 Department of Health Policy and Management, Yale School of Public Health, New Haven, CT 06519, USA; haadia.zaheer@yale.edu

**Keywords:** psychosocial vulnerability, health-related social needs, social determinants of health, cancer surgery, neighborhood characteristics, prehabilitation, preoperative optimization, major surgery

## Abstract

**Background/Objectives**: Psychosocial factors are not routinely screened for during the perioperative period, even though they significantly influence overall health. This study aimed to inventory the psychosocial vulnerabilities among patients undergoing cancer surgery. **Methods**: We conducted a cross-sectional analysis of a researcher-administered psychosocial screener implemented within a statewide health system between July 2023 and August 2025. A 45 min screener was offered to consecutive adult patients within two weeks before their major elective cancer surgery. Residential addresses were geocoded to assign neighborhood deprivation percentiles for the Area Deprivation Index (ADI) and the Social Vulnerability Index (SVI). Elevated psychosocial vulnerability was determined based on a model-based clustering approach, and a high deprivation index was defined as ≥75th percentile. **Results**: A total of 383 patients (37% response rate) completed the screener, including colorectal (40%), thoracic (36%), and surgical oncology (24%) patients, with a median age of 66 years (IQR, 57–73). Over half (52.0%, n = 199) reported ≥2 psychological and ≥2 social vulnerabilities. Younger patients (*p* = 0.021), non-white patients (*p* < 0.001), patients identifying as non-heterosexual (*p* = 0.014), without a partner (*p* < 0.001) or private insurance (*p* = 0.040), and those with lower household income (*p* < 0.001) were more likely to report elevated psychosocial vulnerability. Patients with elevated psychosocial vulnerability were more likely to reside in deprived neighborhoods (ADI: 34.0 vs. 29.0, *p* = 0.035; SVI: 0.35 vs. 0.27, *p* = 0.005). **Conclusions**: Patients undergoing major cancer surgery experience substantial psychosocial vulnerabilities, particularly those from socioeconomically disadvantaged neighborhoods. Future work should identify the psychosocial factors most predictive of poor surgical outcomes to guide targeted preoperative interventions.

## Simple Summary

Psychosocial vulnerabilities are often overlooked in current presurgical optimization programs, despite their growing recognition as a significant factor in outcomes. This study aimed to comprehensively characterize the psychosocial vulnerabilities among patients undergoing major cancer surgery as a critical step toward improving preoperative optimization strategies. This was a cross-sectional analysis of a preoperative psychosocial screener administered to 383 patients across three hospitals, each with three surgical services, within a statewide health system. Across 27 psychosocial domains, more than half of patients with cancer reported at least two psychological vulnerabilities and two social vulnerabilities in the immediate preoperative period. Those residing in more deprived neighborhoods were significantly more likely to report these vulnerabilities, particularly food insecurity and limited health literacy. This preoperative screener provides a model for integrating psychosocial risk assessment into routine surgical care, which could inform targeted preoperative interventions for patients at risk.

## 1. Introduction

Despite advancements in surgical care, postoperative complications are common among patients undergoing cancer surgery [1]. Amid the growing global cancer burden, surgical complications continue to be a source of patient morbidity and negatively impact long-term survival [2,3,4]. Complications that result in a patient’s demise impose significant economic burdens on healthcare systems and payers [5,6]. Surgeons routinely screen for and optimize medical comorbidities before surgery in an effort to reduce postoperative complications [7], and increased healthcare utilization improves outcomes for these patients [8].

However, this same level of heightened attention in the context of psychological and social risk factors has been constrained by limited evidence supporting a similar association with surgical outcomes. While the impact of a cancer diagnosis and surgery on psychosocial health is well documented [9,10,11], emerging evidence highlights a bidirectional causal relationship, where psychosocial health also influences outcomes following cancer surgery. Yet, psychosocial health is often omitted or overlooked in current presurgical optimization programs, though its importance is increasingly recognized [12]. In our group’s prior work, medically comorbid patients with multiple psychosocial risk factors had 3.4-fold higher odds of experiencing a short-term complication following gastrointestinal cancer surgery [13]. Additionally, in patients who underwent major elective surgery across a statewide healthcare system, those with at least one health-related social need (HRSN) had 1.8-fold higher odds [14].

Preoperative psychosocial screeners currently used in clinical settings have been implemented with low fidelity, highlighting the critical need for new strategies to ensure consistent and high-quality psychosocial risk assessment in surgical care [14,15]. Accurately identifying this burden preoperatively will inform shared decision-making, enable targeted interventions for high-risk patients, and ideally improve surgical outcomes [16]. We aimed to comprehensively characterize the psychosocial vulnerabilities among patients undergoing major cancer surgery as a critical step toward improving preoperative optimization strategies. The findings are intended to be a hypothesis-generating study.

## 2. Materials and Methods

### 2.1. Study Design and Ethics Approval

We conducted a cross-sectional analysis of a researcher-administered psychosocial screener implemented across a statewide health system from July 2023 to August 2025. The 45 min screener was offered to consecutive adult patients within two weeks before their major elective cancer surgery at three hospitals within the system. The study was conducted in accordance with the Declaration of Helsinki and was approved by the Yale University Institutional Review Board (#2000031799). Informed verbal consent was obtained from all subjects involved.

### 2.2. Inclusion and Exclusion Criteria

We invited consecutive adult patients with a known or suspected cancer diagnosis undergoing major elective curative-intent surgery to participate. Patients receiving surgical care across thoracic (six surgeons), surgical oncology (hepatopancreatobilary, peritoneal surface malignancies, and sarcoma; four surgeons), and colorectal surgery (six surgeons) services were included. Major surgery was defined as the removal of a tumor within the thoracic or abdominal cavity via a transthoracic or transabdominal approach. Open, laparoscopic, and robotic approaches were included. Ostomy creation alone and transanal surgeries were excluded. Patients under legal conservatorship and non-English-speaking patients were excluded. Future screenings were translated into the second most common language spoken by patients (Spanish), but that subgroup of the registry has not yet completed accrual.

Two neighborhood-level deprivation indices, the Area Deprivation Index (ADI) and the Social Vulnerability Index (SVI), were derived from 2020 U.S. Census data. These indices are well validated and have been described previously [17,18,19]. National-level percentile data was used for both indices. Residential addresses from the electronic health record were geocoded to the census tract level and block group level for the Social Vulnerability Index and Area Deprivation Index, respectively. Higher percentiles on these measures indicate greater neighborhood deprivation. Patients with missing or non-geocodable addresses were excluded from the analysis. Patients with non-numerical ADI values were also excluded to maintain interpretability and comparability of deprivation percentiles.

### 2.3. Preoperative Psychosocial Screener

The preoperative psychosocial screener is a comprehensive, researcher-administered instrument designed to assess psychosocial risk across 27 domains, encompassing 136 items within 10 psychological and 17 social domains. Each domain consists of a scale adapted from a previously validated instrument. The social risk screener was adapted from the Socioecological Determinants of Health-88 (SEDOH-88), an 88-question survey across 31 SEDOH domains [20]. The Supplemental Methods S2 details each psychosocial domain, the items within each domain, the corresponding previously validated instrument, and the scoring system. Patient responses to the preoperative psychosocial screen were used to calculate an overall psychosocial vulnerability score. A higher score indicates greater psychosocial vulnerability.

Face and content validity were achieved through expert review, iterative refinement during instrument development, and pilot testing. Experts evaluated the comprehensiveness of the instrument, the relevance of its incorporated scales, and the clarity and appropriateness of the individual items. Feedback informed iterative refinement throughout instrument development and pilot testing. The preoperative psychosocial screen was administered over the telephone by trained researchers (K.S., S.L., E.P., E.E., B.T.B., Y.H., and H.Z.) within two weeks before the patient’s scheduled surgery. The survey took approximately 45 min to complete. Only patients who completed the entire survey were included in this study. We prespecified exclusion of participants who elected to complete only a subset of the screener (e.g., the behavioral domains) from this analysis, as it would have required substantial imputation across multiple psychosocial domains to derive their overall vulnerability score.

### 2.4. Sociodemographic and Clinical Covariates of Interest

In addition to structural characteristics, psychosocial vulnerability was analyzed across key sociodemographic and clinical characteristics, including gender, race, ethnicity, socioeconomic status (i.e., income level), and primary surgical service. Gender, race, ethnicity, and income level were self-reported and treated as binary variables. All patients in this cohort identified as either male or female, so gender was analyzed as a binary construct. Income was categorized based on the U.S. Federal Poverty Line (FPL), adjusted for household size, number of children under 18, and the age of the householder. Low income was defined as an annual income below the U.S. FPL, middle income as between 100% and 200% of the FPL, and high income as above 200%. Definitions for other demographic variables are provided in the Supplemental Methods S1.

Clinical variables, including the presence of high-risk medical comorbidities and the patient’s primary surgical service, were extracted from the electronic medical record (EMR) by one researcher and independently verified by another. High-risk comorbidity status was coded as a binary variable, with patients classified as high-risk if they had at least one of the following conditions: body mass index (BMI) ≥ 35, diabetes, functional status, chronic obstructive pulmonary disease (COPD), ascites, heart failure, disseminated cancer, immunotherapy, bleeding disorder, recent blood transfusion (within 72 h before surgery), sepsis, oxygen support, ventilator dependence, dialysis, recent fall (within six months), or dementia.

### 2.5. Statistical Analyses

Study data was collected and managed using REDCap electronic data capture tools hosted at Yale University (New Haven, CT, USA) [21,22]. Statistical analyses were performed using R version 4.5.1 (R Core Team, 2025; R Foundation for Statistical Computing, Vienna, Austria) and the following statistical packages: tidyverse, lubridate, janitor, mclust, and stats. As this was a descriptive, exploratory study, no formal sample size or power calculation was performed. The study was designed to capture real-world data using a consecutive sampling approach, and the number of eligible patients determined the sample size during the study period. The goal was not hypothesis testing but rather to describe patterns and generate insights to inform future hypothesis-driven work. While this was an exploratory study and the goal was not hypothesis testing, we performed statistical comparisons across sociodemographic groups to identify potential patterns in preoperative psychosocial burden and generate insights to inform future hypothesis-driven work.

We categorized the overall psychosocial vulnerability score into two groups using a model-based clustering approach. Specifically, we fitted a univariate Gaussian finite-mixture model with two components to the score distribution, applying a loosened cutoff (P[high] ≥ 0.35) to identify two latent groups. These groups were labeled “Elevated Psychosocial Risk” and “Limited Psychosocial Risk.” This data-driven approach was chosen to identify natural groupings in the score distribution, rather than relying on an arbitrary cutoff. Individual psychosocial domains were also assessed to identify specific patterns of vulnerability. The Supplemental Methods S3 describes the operationalization of these domains into binary variables. Neighborhood deprivation was quantified using national percentiles of the ADI and SVI and analyzed both as a continuous measure and as a binary variable, with high deprivation defined as the 75th percentile or higher. This threshold was chosen to maximize contrast between groups while avoiding restriction to only the extreme tail of the distribution.

Demographic and clinical characteristics were summarized using descriptive statistics and stratified by covariates of interest. The Shapiro–Wilk test was used to assess the normality of the overall preoperative psychosocial score. If normality was not met, we planned to proceed with nonparametric tests as follows. Categorical variables would be reported as percentages and compared using Chi-squared or Fisher’s exact tests. Continuous variables would be reported as medians and interquartile ranges (IQRs) and compared using Wilcoxon rank-sum tests. To account for multiple testing across 27 psychosocial domains, a Benjamini–Hochberg (BH) correction was applied to control the false-discovery rate while maintaining statistical power (“q-value”) [23]. For comparisons by income group and surgical service, post hoc pairwise analyses were conducted using pairwise Chi-squared or Fisher’s exact tests with BH correction following a significant overall BH-adjusted test. Findings were reported according to the guidelines established by the Strengthening the Reporting of Observational Studies in Epidemiology (STROBE) statement.

## 3. Results

### 3.1. Demographics and Overall Preoperative Psychosocial Vulnerability Score

**Figure 1:**
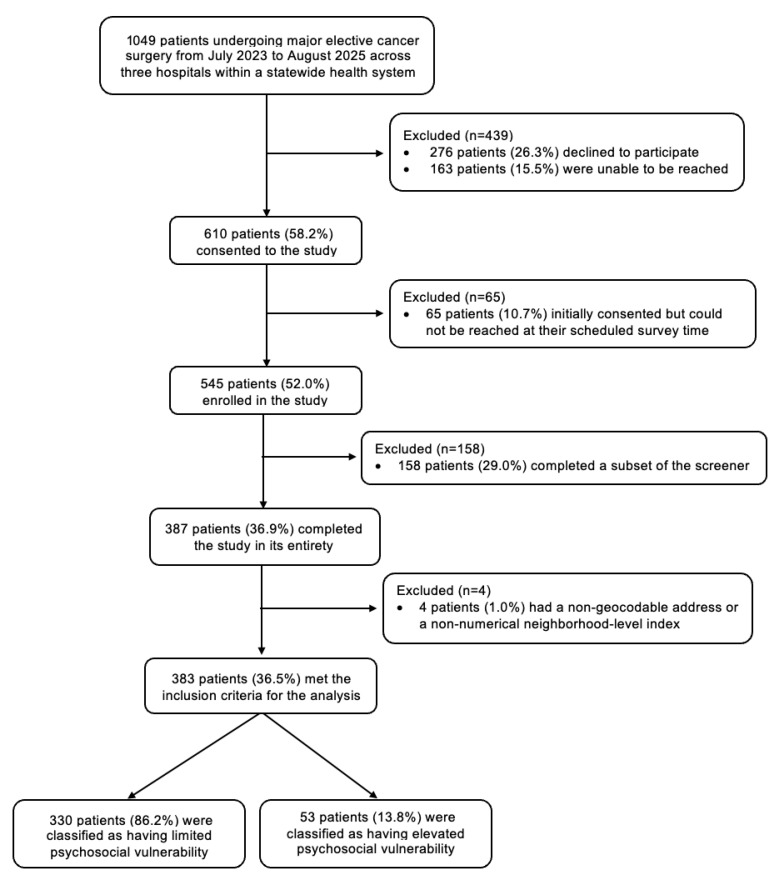
Flow diagram of the study population.

Of the 1049 patients who met the inclusion criteria, 545 (52.0%) enrolled in the study, and 387 (response rate: 36.5%) completed the preoperative psychosocial screen in its entirety (Figure 1). Four patients were excluded because their residential address geocoded to a non-numeric ADI percentile (i.e., GQ or PH). Non-respondents were more likely to be non-white, Hispanic, and live in a neighborhood with higher deprivation (Supplemental Table S1). Of the 383 patients who met the inclusion criteria for analysis, the median age was 66 years (IQR, 57–73), 50% (n = 193) identified as female, 8.6% (n = 33) as non-white, 5.2% (n = 20) as Hispanic, and 4.2% (n = 16) as non-heterosexual. One (0.3%) patient was a refugee, and two (0.5%) patients had been incarcerated in the past year. Thirty-four (8.9%) patients had served in the U.S. armed forces, including five with combat service and one currently on active duty.

The median score on the survey was 7 (IQR, 5–11), with a range of 0 to 38. Nearly all patients (94.8%, n = 363) reported at least two psychosocial vulnerabilities, and one-third (30.8%, n = 118) reported at least one need of four core social domains (housing, transportation, food, or utilities). In total, 52.0% (n = 199) of patients reported two or more psychological vulnerabilities and two or more social vulnerabilities. The overall preoperative psychosocial vulnerability score was non-normally distributed (Shapiro–Wilk test, *p* < 0.001), supporting the use of a model-based clustering approach to identify risk groups.

**Figure 2:**
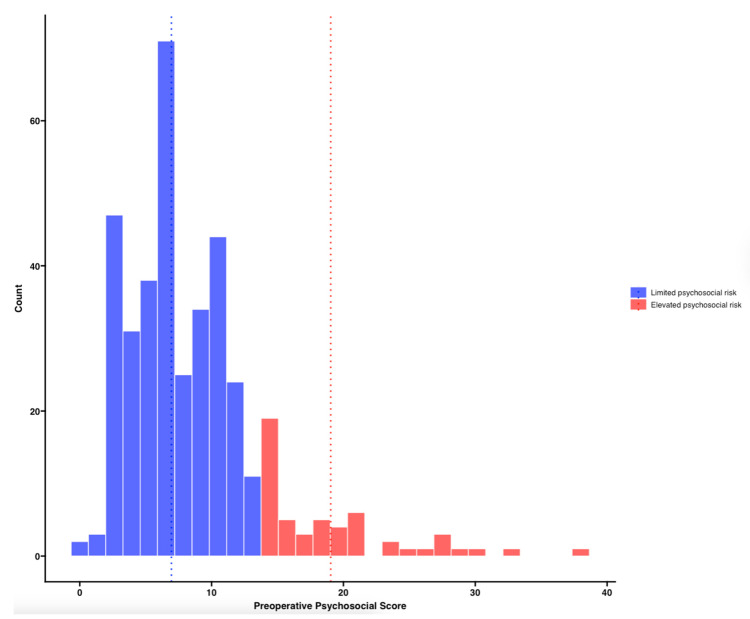
Histogram of patient scores on the preoperative psychosocial screener, classified using a Gaussian mixture model with a loosened cutoff (P[high] ≥ 0.35). Patients were grouped into elevated psychosocial risk (red) and limited psychosocial risk (blue) based on their posterior probability of group membership. Vertical dashed lines indicate the mean score within each group.

Using a Gaussian mixture model, patients were grouped into elevated psychosocial risk (n = 53, 13.8%) and limited psychosocial risk (n = 330, 86.2%; Figure 2). Elevated psychosocial vulnerability in the immediate preoperative period was significantly more common among younger patients (*p* = 0.021), patients who identified as non-white (*p* < 0.001), patients with a sexual identity other than heterosexual (*p* = 0.014), non-partnered patients (*p* < 0.001), patients without private insurance (*p* = 0.040), and in those whose household income fell below 200% of the FPL (*p* < 0.001; Table 1).

### 3.2. Psychosocial Vulnerability Across Primary Surgical Services

One hundred fifty-three (40%) patients were treated by the colorectal surgery services, 137 (36%) by the thoracic surgery services, and 93 (24%) by the surgical oncology services. Psychosocial vulnerability was consistent across primary surgical services, except for a history of tobacco use (q < 0.001) (Supplemental Table S2). In post hoc pairwise tests, a history of tobacco use was more common among patients treated by thoracic surgery (71%) compared to colorectal surgery (41%; q < 0.001) and surgical oncology (41%; q < 0.001; Supplemental Table S3).

### 3.3. Relationship Between Patient- and Neighborhood-Level Vulnerability

The median ADI percentile was 30.0 (IQR: 20.0–43.0; range: 1–99), and the median SVI percentile was 0.28 (IQR: 0.13–0.52; range: 0.001–0.991). Patients with elevated psychosocial vulnerability were more likely to reside in more deprived neighborhoods than those with limited vulnerability, as indicated by the higher ADI percentile (median [IQR]: 34.0 [23.0–50.0] vs. 29.0 [20.0–43.0], *p* = 0.035, respectively) and higher overall SVI percentile (median [IQR]: 0.35 [0.23–0.82] vs. 0.27 [0.11–0.51], *p* = 0.005). Adjusting for multiple comparisons, this association with the SVI remained significant for three of its four subthemes (Table 2).

**Figure 3:**
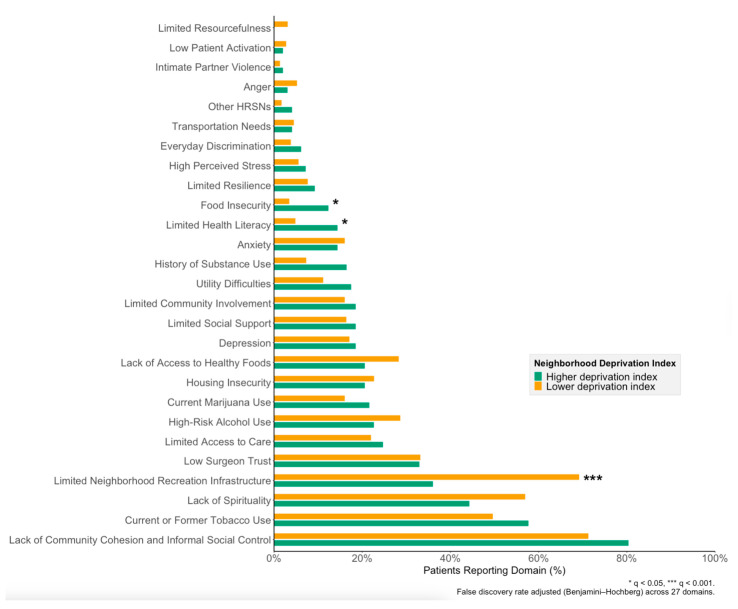
Psychological and social domains reported by patients undergoing major elective cancer surgery, stratified by Social Vulnerability Index (SVI). Residential addresses were geocoded to the census tract level to determine each neighborhood’s national SVI percentile, with high deprivation defined as ≥75th percentile. To account for multiple comparisons across 27 psychosocial domains, a Benjamini–Hochberg correction was applied to control the false-discovery rate while maintaining statistical power. Reported q-values reflect the adjusted significance levels from Chi-squared or Fisher’s exact tests. Abbreviations: SVI, Social Vulnerability Index; BH, Benjamini–Hochberg; FDR, false-discovery rate.

Based on the ADI, high deprivation was associated with food insecurity (14% vs. 2.5%, *p* < 0.001) but also with greater neighborhood recreation infrastructure (52% vs. 34%, *p* = 0.023; Supplemental Table S4). Using the overall SVI percentile, higher neighborhood deprivation was similarly associated with greater patient-reported food insecurity (12% vs. 3.5%, *p* = 0.034), limited health literacy (14% vs. 4.9%, *p* = 0.034), and greater neighborhood recreation infrastructure (64% vs. 31%, *p* < 0.001; Figure 3).

### 3.4. Psychosocial Vulnerability by Self-Identified Gender, Race, and Ethnicity

Few psychological and social vulnerabilities differed by self-identified gender (Supplemental Table S5. Men were more likely to report a lack of spirituality or religious affiliation (65% vs. 43%, q < 0.001) and limited community involvement than women (23% vs. 11%, q = 0.044). Patients identifying as Hispanic and/or non-white were considerably more likely than non-Hispanic white patients to report food insecurity (21% vs. 3.6%, q = 0.002) but less likely to report limited neighborhood recreation infrastructure (36% vs. 64%, q = 0.005; Supplemental Table S6).

### 3.5. Psychosocial Vulnerability by Socioeconomic Status

Psychosocial vulnerabilities varied across income groups among patients undergoing major cancer surgery, with notable differences mainly in social domains (Table 3). There were differences in food insecurity (q = 0.001), transportation needs (q = 0.017), utility difficulties (q = 0.003), intimate partner violence (q = 0.001), limited social support (q = 0.021), limited healthcare access (q = 0.017), low patient activation (q = 0.006), limited health literacy (q = 0.003), and other HRSNs (q = 0.006) across the income groups. In post hoc pairwise comparisons, patients with low income were more likely to report higher rates of food insecurity (23% vs. 2.9%, q = 0.019) and utility difficulties (38% vs. 9.4%, q = 0.03) compared to those with high income. They also experienced lower patient activation compared to patients with middle income (23% vs. 0%, q = 0.047) and high income (23% vs. 1.6%, q = 0.016). Patients with middle income reported lower health literacy compared to those with high income (19% vs. 4.5%, q = 0.018); Supplemental Table S7). There were also differences in limited resourcefulness (q = 0.004), anger (q = 0.003), and history of SUD (q = 0.023) across the income groups. In post hoc pairwise comparisons, patients with low income were more likely to report limited resourcefulness compared to patients with middle income (23% vs. 0%, q = 0.047) and high income (23% vs. 1.3%, q = 0.010). 

## 4. Discussion

This study aimed to evaluate the preoperative psychosocial vulnerabilities among patients undergoing major elective cancer surgery. We conducted a cross-sectional analysis using a prospectively maintained registry across three hospitals, each with three surgical services, within an integrated healthcare system. Our findings revealed substantial psychosocial vulnerabilities in this population, as over half of the patients reported two or more psychological and two or more social vulnerabilities across 27 assessed domains. The burden of psychosocial vulnerability among patients undergoing cancer surgery in our study is comparable to that reported in a researcher-administered survey of patients at a Mid-Atlantic tertiary academic center. That cohort had a similar age and sex distribution, but with a higher proportion of non-white patients, and found that 73% of patients had at least one psychosocial vulnerability [13].

Patients living in more deprived neighborhoods were more likely to report elevated psychosocial vulnerability. This finding supports the convergent validity of the preoperative psychosocial screener with established geography-based research tools. However, most individual domains captured by the patient-level survey were not significantly associated with neighborhood deprivation. Because neighborhood-level indices are readily available, they are often used as proxies for individual-level vulnerability [24]. However, we observed discrepancies between these multilevel measures. Our data supports the concept of ecological fallacy, a measurement error that occurs when group-level associations are applied inappropriately to individuals [25]. Despite these limitations, two patient-level domains, food insecurity and health literacy, were significantly associated with neighborhood deprivation based on the SVI. These domains are not only modifiable but have also been linked to adverse surgical outcomes across multiple specialties, suggesting that they may be particularly valuable targets for multilevel interventions [26,27,28]. Investigation into the impact of food assistance, whether through the Supplemental Nutritional Assistance Program or unconditional cash transfers, on surgical patients experiencing food insecurity is warranted [29,30]. In addition, the potential of targeted interventions to address low health literacy as a means to improve surgical outcomes merits consideration [31].

While our study highlights the limitations of neighborhood indices, it also reveals how the use of only patient-level data can obscure geographic inequities. For example, patients from more deprived neighborhoods reported greater access to neighborhood recreation infrastructure compared to those in less deprived areas, a finding that initially appears counterintuitive. However, a closer examination of the relevant survey items suggests they may assess walkability rather than actual infrastructure, potentially favoring urban environments and introducing bias against suburban and rural settings [32,33]. A similar pattern was observed in the domain related to food access. Whereas our study was conducted at three urban hospitals in Connecticut, the Chu Lab (Birmingham, AL, USA), developers of the SEDOH-88, administered the survey to patients at three rural hospitals in Alabama, where most patients were screened before their colonoscopy [34]. Their cohort was similar in age but had a higher proportion of non-white patients and lived in neighborhoods with markedly higher deprivation percentiles. Yet, patient-reported neighborhood recreation infrastructure and access to healthy foods closely mirrored those reported by our patients living in less deprived areas. This contrast, between a cohort with fewer minorities in more urban, less deprived neighborhoods and a cohort with more minorities in more rural, highly deprived neighborhoods, suggests that some aspects of the preoperative psychosocial screener may require revision or contextual adaptation for geographically diverse populations. While neighborhood-level measures could be relied upon for these domains [35], they are also prone to these biases [36,37]. To improve interpretability, researchers should consider incorporating additional contextual measures, such as rurality indices or built environment metrics [38]. Applying spatial analysis techniques could also provide richer insights into the interaction of the SVI, the ADI, and patient-level vulnerabilities.

Contrary to expectations, we found that psychosocial vulnerability did not exhibit consistent patterns across common disparity markers, such as gender, race, ethnicity, and socioeconomic status. Although disparities in surgical outcomes are well-documented across fixed sociodemographic characteristics [39], our study presents a range of potentially modifiable psychosocial domains that do not align neatly with these traditional axes. This suggests that psychosocial risk may represent a distinct dimension of vulnerability. A critical next step is to evaluate the true modifiability and clinical impact of these domains in relation to surgical outcomes. Structural equation modeling that incorporates fixed characteristics and actionable psychosocial factors could offer deeper insight into surgical disparities and reveal novel targets for intervention [40].

Medical risk stratification and optimization before surgery is a well-established and cost-effective mechanism for improving outcomes in patients with significant medical comorbidities [8,41]. While surgeons are often cautious about performing elective surgery on medically complex patients without evaluation and management of their medical comorbidities, a similar level of wariness is not typically applied to psychosocial risk factors [42]. However, there is growing recognition of the importance of identifying and mitigating these modifiable risk factors [43], with patients demonstrating a general acceptance of screening practices [44]. Over the last 50 years, non-surgical research has consistently shown that psychosocial risk factors contribute to poorer outcomes for individuals with chronic diseases, and that interventions targeting modifiable behavioral, cognitive, and social factors yield more favorable outcomes [45]. Undergoing major cancer surgery demands physical, mental, emotional, and social resilience, and, akin to training for a marathon, patients could train for the complex and multifaceted journey of surgery [46]. Surgery presents a unique chance to improve the health of patients experiencing psychosocial vulnerabilities, as this acute life stressor can lead to lasting health behavior changes [47]. Therefore, the perioperative period presents an ideal time to screen for and address psychosocial risks [48,49], and previous work has demonstrated meaningful improvements in surgical outcomes and cost savings from prehabilitation programs, which include stress reduction techniques [50]. However, current surgical risk prediction models (e.g., American College of Surgeons’ National Surgical Quality Improvement Program Risk Calculator) do not include psychological and social risk factors [51]. Our preoperative psychosocial screener aims to address this identified gap in formal psychosocial health screening within standard surgical practice.

This study aligns with prior evidence suggesting that existing screening procedures may underestimate the true extent of psychosocial risk in this patient population [52]. In abdominal cancer surgery, Meyers et al. found a high level of discordance between routine documentation of psychosocial domains and a formal screening measure [15]. In our group’s previous study of a bedside nursing-administered screener, only 6% of patients undergoing major surgery reported at least one of four social domains [14]. The present study, conducted within the same healthcare system, found that 31% of patients reported at least one of the four social domains on our researcher-administered screener. Our previous study included patients undergoing surgery for benign conditions; however, this discrepancy suggests that capturing social needs may depend on the interviewer and the interview setting. An analysis of 651 community health centers in 21 U.S. states noted differences in patient reports of social risks across racial, ethnic, and language groups [53]. Interviewer error is a well-described phenomenon in public health surveys [54]. Future studies should investigate how the interviewer–interviewee dyad, as well as the interview setting, might impact the quality and validity of social data collected.

This study has limitations that impact how we interpret our findings. First, the non-response rate was 63%, which could lead to non-response bias. To minimize this bias, we contacted patients up to three times at different times of day and on various dates before being marked as unreachable. Because the survey took 45 min to complete, it is understandable that one in four patients declined to participate, and nearly one in three who initially agreed did not finish the study. Patients from racially marginalized groups, Hispanic patients, and those living in higher-deprivation neighborhoods were less likely to complete the study, potentially limiting the external validity of our results for these populations. This presents an opportunity to adopt community-engaged principles to target non-respondents and to apply dimensionality reduction techniques to enhance the survey’s efficiency and clinical usefulness [55]. Patients with psychosocial vulnerabilities are also less likely to complete surveys [56,57]. A strength of this study was the use of researcher-administered, multi-dimensional surveys instead of asynchronous, self-paced surveys, as data suggests that the former tend to have higher completion rates [58]. While item non-response is higher in self-administered surveys, social desirability bias is higher in interview modes. All survey interviews were conducted by trainees affiliated with an academic institution, which may have introduced social desirability bias [59]. This limitation emphasizes the importance of integrating community-engaged principles into psychosocial screening to foster trust and minimize measurement error [60]. While we used model-based practices to determine the cutoff for overall psychosocial vulnerability, future studies should assess how alternative approaches (e.g., κ-means clustering) might influence the results. Finally, although our preoperative psychosocial screener is composed of previously developed and validated instruments, ongoing psychometric testing is necessary to confirm its validity and reliability, particularly that of the overall psychosocial score, in this patient population. Incorporating mixed-effects models with surgical service or cancer type as a random effect could yield more accurate and reliable estimates by accounting for clustering. Together, these steps would support the screener’s adoption in real-world settings.

## 5. Conclusions

Our study reveals substantial psychosocial vulnerabilities among patients scheduled for major elective cancer surgery, without a consistent pattern across traditional sociodemographic markers of disparity. Patients living in more deprived neighborhoods were significantly more likely to report psychosocial vulnerabilities. Notably, this association persisted for food insecurity and limited health literacy but was not observed across most individual domains. Our preoperative screener offers a potential model for integrating psychosocial risk assessment into routine surgical care. Future research should focus on optimizing the screener’s structure, for instance by applying item response theory. It should also identify the psychological and social domains most predictive of adverse surgical outcomes to inform targeted preoperative interventions.

## Figures and Tables

**Table 1:** Demographics and clinical characteristics of patients with cancer undergoing major elective surgery across three hospitals, each with three surgical services, within a statewide health system from July 2023 to August 2025. Characteristics are categorized by self-reported psychosocial vulnerability across 27 domains.

**Characteristics, n (%)**	**Overall** **(n = 383)**	**Limited Psychosocial Vulnerability** **(n = 330)**	**Elevated Psychosocial Vulnerability ^a^** **(n = 53)**	***p*-Value**
Age, years, (median, IQR)	66 (57–73)	66 (58–73)	63 (51–70)	0.021
Sex assigned at birth				0.568
Male	191 (50)	167 (51)	24 (45)	
Female	192 (50)	163 (49)	29 (55)	
Self-identified gender				0.596
Man	190 (50)	166 (50)	24 (45)	
Woman	193 (50)	164 (50)	29 (55)	
Self-identified race				<0.001
White	350 (91)	310 (94)	40 (75)	
Non-white	33 (8.6)	20 (6.1)	13 (25)	
Self-identified ethnicity				0.500
Non-Hispanic	363 (95)	314 (95)	49 (92)	
Hispanic	20 (5.2)	16 (4.8)	4 (7.5)	
Sexual identity				0.014
Heterosexual	367 (96)	320 (97)	47 (89)	
Non-heterosexual	16 (4.2)	10 (3.0)	6 (11)	
Marital status				<0.001
Partnered	252 (66)	230 (70)	22 (42)	
Non-partnered	131 (34)	100 (30)	31 (58)	
Education				0.057
<Four-year college degree	167 (44)	137 (42)	30 (57)	
≥Four-year college degree	216 (56)	193 (58)	23 (43)	
Employment status				0.152
Employed	161 (42)	144 (44)	17 (32)	
Not employed	222 (58)	186 (56)	36 (68)	
Primary insurance coverage				0.040
Government	224 (58)	189 (57)	35 (66)	
Private insurance	155 (40)	139 (42)	16 (30)	
Uninsured	4 (1.0)	2 (0.6)	2 (3.8)	
Socioeconomic status ^b^				<0.001
<U.S. FPL	13 (3.4)	7 (2.1)	6 (11)	
100–200% U.S. FPL	36 (9.4)	30 (9.1)	6 (11)	
≥200% U.S. FPL	308 (80)	276 (84)	32 (60)	
Unknown	26 (6.8)	17 (5.2)	9 (17)	
High-risk medical comorbidity ^c^				
≥1 comorbidity	190 (50)	158 (48)	32 (60)	0.123
Number of high-risk medical comorbidities				0.323
0	193 (50)	172 (52)	21 (40)	
1	110 (29)	90 (27)	20 (38)	
2	45 (12)	39 (12)	6 (11)	
3+	35 (9.1)	29 (8.8)	6 (11)	
Primary surgical service				0.449
Thoracic	137 (36)	119 (36)	18 (34)	
Surgical oncology	93 (24)	83 (25)	10 (19)	
Colorectal	153 (40)	128 (39)	25 (47)	

Percentages might not add to 100% due to rounding. The Wilcoxon rank-sum test was used for continuous variables. Chi-squared and Fisher’s exact tests were used for categorical variables, as appropriate. ^a^ Elevated psychosocial vulnerability was defined as a score of ≥75th percentile on the preoperative psychosocial screener. ^b^ The Federal Poverty Line threshold was adjusted for household size, number of children under 18, and the age of the householder. ^c^ The definition of a high-risk medical comorbidity is outlined in Section 2 of the manuscript. The definitions of marital status, education, employment status, primary insurance coverage, and socioeconomic status are outlined in the Supplemental Methods S1. Abbreviations: IQR, interquartile range; U.S., United States; FPL, federal poverty line.

**Table 2:** Neighborhood-level indices of patients undergoing major cancer surgery across three hospitals, each with three surgical services, within a statewide health system from July 2023 to August 2025, stratified by patient-level psychosocial vulnerability.

**Neighborhood-Level Indices, Median (IQR)**	**Overall** **(n = 383)**	**Limited Psychosocial Vulnerability** **(n = 330)**	**Elevated Psychosocial Vulnerability** **(n = 53)**	***p*-Value**	q-Value *
ADI percentile	30.0 (20.0–43.0)	29.00 (20.0–43.0)	34.0 (23.0–50.0)	0.035	---
SVI percentile (overall)	0.28 (0.13–0.52)	0.27 (0.11–0.51)	0.35 (0.23–0.82)	0.005	---
Theme 1—Socioeconomic Status	0.26 (0.13–0.50)	0.24 (0.13–0.46)	0.38 (0.17–0.75)	0.005	0.008
Theme 2—Household Composition and Disability	0.39 (0.22–0.63)	0.38 (0.20–0.60)	0.49 (0.35–0.77)	0.006	0.008
Theme 3—Minority Status and Language	0.33 (0.19–0.52)	0.32 (0.19–0.49)	0.41 (0.27–0.71)	0.001	0.005
Theme 4—Housing Type and Transportation	0.34 (0.15–0.57)	0.34 (0.15–0.56)	0.38 (0.20–0.73)	0.211	0.211

The national deprivation percentile was used for both neighborhood-level indices. Patients with non-numerical ADI ranks were excluded to maintain interpretability and comparability of deprivation percentiles. * To account for multiple comparisons across the four SVI subthemes, a Benjamini–Hochberg correction was applied to control the false-discovery rate while maintaining statistical power. Reported q-values reflect the adjusted significance levels from Wilcoxon rank-sum tests. Abbreviations: ADI, Area Deprivation Index; SVI, Social Vulnerability Index; IQR, interquartile range.

**Table 3:** Psychological and social vulnerabilities reported by patients undergoing major cancer surgery, stratified by income.

**Psychosocial Domains, n (%)**	**Overall** **(n = 383)**	**Low** **Income** **(n = 13)**	**Middle** **Income** **(n = 36)**	**High** **Income** **(n = 308)**	Unknown * (n = 26)	***p*-Value**	**q-Value ^†^**
Psychological domains							
≥Moderate anxiety	60 (16)	4 (31)	3 (8.3)	48 (16)	5 (19)	0.239	0.293
≥Moderate depression	67 (17)	5 (38)	6 (17)	49 (16)	7 (27)	0.097	0.164
Lack of spirituality/religion	206 (54)	3 (23)	17 (47)	173 (56)	13 (50)	0.093	0.164
Low resilience	31 (8.1)	2 (15)	3 (8.3)	22 (7.1)	4 (15)	0.250	0.293
Limited resourcefulness	9 (2.3)	3 (23)	0 (0)	4 (1.3)	2 (7.7)	<0.001	0.004
Anger	18 (4.7)	2 (15)	0 (0)	10 (3.2)	6 (23)	<0.001	0.003
High-risk alcohol use	104 (27)	0 (0)	8 (22)	89 (29)	7 (27)	0.124	0.186
History of tobacco use	198 (52)	6 (46)	22 (61)	154 (50)	16 (62)	0.423	0.431
Current marijuana use	67 (17)	3 (23)	3 (8.3)	56 (18)	5 (19)	0.431	0.431
History of SUD	37 (9.7)	4 (31)	7 (19)	24 (7.8)	2 (7.7)	0.010	0.023
Social domains							
Food insecurity	22 (5.7)	3 (23)	5 (14)	9 (2.9)	5 (19)	<0.001	0.001
Transportation needs	17 (4.4)	2 (15)	2 (5.6)	9 (2.9)	4 (15)	0.006	0.017
Housing insecurity	85 (22)	5 (38)	11 (31)	61 (20)	8 (31)	0.134	0.191
Utility difficulties	49 (13)	5 (38)	9 (25)	29 (9.4)	6 (23)	<0.001	0.003
Intimate partner violence	6 (1.6)	1 (7.7)	0 (0)	1 (0.3)	4 (15)	<0.001	0.001
Limited social support	65 (17)	5 (38)	9 (25)	43 (14)	8 (31)	0.009	0.021
Limited access to care	87 (23)	2 (15)	9 (25)	63 (20)	13 (50)	0.006	0.017
Low patient activation	10 (2.6)	3 (23)	0 (0)	5 (1.6)	2 (7.7)	0.002	0.006
Limited health literacy	28 (7.3)	3 (23)	7 (19)	14 (4.5)	4 (15)	<0.001	0.003
High perceived stress	23 (6.0)	3 (23)	2 (5.6)	15 (4.9)	3 (12)	0.031	0.064
Limited community involvement	64 (17)	5 (38)	7 (19)	47 (15)	5 (19)	0.148	0.200
Limited surgeon trust	127 (33)	7 (54)	10 (28)	100 (32)	10 (38)	0.337	0.379
Everyday discrimination	17 (4.4)	2 (15)	3 (8.3)	12 (3.9)	0 (0)	0.076	0.146
Lack of access to healthy foods	101 (26)	2 (15)	10 (28)	87 (28)	2 (7.7)	0.108	0.172
Limited neighborhood recreation infrastructure	233 (61)	6 (46)	17 (47)	195 (63)	15 (58)	0.181	0.233
Lack of community cohesion and informal social control	282 (74)	12 (92)	28 (78)	222 (72)	20 (77)	0.364	0.394
Other HRSNs ^‡^	9 (2.3)	2 (15)	2 (5.6)	3 (1.0)	2 (7.7)	0.002	0.006

Domains, items, instruments, and scoring are listed in Supplemental Methods S2; binary operationalization is described in Supplemental Methods S3. Income was categorized based on the U.S. Federal Poverty Line (FPL), adjusted for household size, number of children under 18, and the age of the householder. Low income was defined as an annual income below the U.S. FPL, middle income as between 100% and 200% of the FPL, and high income as above 200%. * Patients who did not know their annual household income or declined to answer were categorized as “unknown.” ^†^ To account for multiple comparisons across the 27 psychosocial domains, a Benjamini–Hochberg correction was applied to control the false-discovery rate while maintaining statistical power. Reported q-values reflect the adjusted significance levels. ^‡^ Other HRSNs included clothing, childcare, and other self-reported social needs within the past 12 months. Abbreviations: HRSNs, health-related social needs; SUD, substance use disorder; FPL, federal poverty line.

## Data Availability

The raw data supporting the conclusions of this article will be made available by the authors on request.

## Supplementary Materials

The following supporting information can be downloaded at: https://www.mdpi.com/article/10.3390/cancers17172859/s1, Supplemental Methods S1: Definitions for selected demographic variable categories. Supplemental Methods S2: The preoperative psychosocial screener is a comprehensive, patient-reported assessment tool covering 27 psychosocial domains. Supplemental Methods S3: Operationalizing the individual psychosocial domains. Supplemental Table S1: Demographics, neighborhood deprivation index, and clinical characteristics of survey respondents, partial respondents, and non-respondents. Supplemental Table S2: Psychological and social vulnerabilities reported by patients undergoing major elective cancer surgery, stratified by primary surgical services. Supplemental Table S3: Post-hoc pairwise comparisons of psychological and social vulnerabilities by primary surgery service. Supplemental Table S4: Psychological and social vulnerabilities reported by patients undergoing major elective cancer surgery, stratified by Area Deprivation Index. Supplemental Table S5: Psychological and social vulnerabilities reported by patients undergoing major elective cancer surgery, stratified by self-identified gender. Supplemental Table S6: Psychological and social vulnerabilities reported by patients undergoing major elective cancer surgery, stratified by self-identified race and ethnicity. Supplemental Table S7: Post-hoc pairwise comparisons of psychological and social vulnerabilities by household income. References [61,62,63,64,65,66,67,68,69,70,71,72,73,74,75,76,77,78,79,80,81,82,83,84] are cited in the supplementary materials.

## Abbreviations

The following abbreviations are used in this manuscript:

HRSN	Health-related social need
SEDOH-88	Socioecological Determinants of Health-88
ADI	Area Deprivation Index
SVI	Social Vulnerability Index
FPL	Federal Poverty Line
EMR	Electronic medical record
REDCap	Research Electronic Data Capture
STROBE	Strengthening the Reporting of Observational Studies in Epidemiology
BMI	Body mass index
COPD	Chronic obstructive pulmonary disease
SUD	Substance use disorder
IQR	Interquartile range
U.S.	United States
