# Supplementary material for: Insult to Injury: Cross-Sectional Analysis of Preoperative Psychosocial Vulnerabilities in Adult Patients Undergoing Major Elective Cancer Surgery
Source: Cancers (Basel). 2025 Aug 30;17(17):2859. doi: 10.3390/cancers17172859 (PMC12427204; doi:10.3390/cancers17172859)
Supplement: Supplementary file 1 [file cancers-17-02859-s001.zip › Cancers_SupplementalTables_20250827.pdf]

SUPPLEMENTAL TABLES

Supplemental Table S1. Demographics, neighborhood deprivation index, and clinical characteristics of survey respondents, partial respondents, and non-respondents.

Supplemental Table S2. Psychological and social vulnerabilities reported by patients undergoing major elective cancer surgery, stratified by primary surgical services.

Supplemental Table S3. Post-hoc pairwise comparisons of psychological and social vulnerabilities by primary surgery service.

Supplemental Table S4. Psychological and social vulnerabilities reported by patients undergoing major elective cancer surgery, stratified by Area Deprivation Index.

Supplemental Table S5. Psychological and social vulnerabilities reported by patients undergoing major elective cancer surgery, stratified by self-identified gender.

Supplemental Table S6. Psychological and social vulnerabilities reported by patients undergoing major elective cancer surgery, stratified by self-identified race and ethnicity.

Supplemental Table S7. Post-hoc pairwise comparisons of psychological and social vulnerabilities by household income.

Supplemental Table S1. Demographics, neighborhood deprivation index, and clinical characteristics of survey respondents, partial respondents, and non-respondents.

**Characteristics, n (%)**	Respondents (n=383)	Partial Respondents (n=132)	Non-Respondents* (n=534)	p-value
Age, years, (median, IQR)	66 (57-73)	65 (56-71)	66 (57-74)	0.244
Sex assigned at birth				0.589
Male	191 (50%)	59 (45%)	259 (49%)	
Female	192 (50%)	73 (55%)	272 (51%)	
Gender				0.617
Man	190 (50%)	59 (45%)	259 (49%)	
Woman	193 (50%)	73 (55%)	272 (51%)	
Race				<0.001
White	350 (91%)	102 (86%)	385 (81%)	
Non-white	33 (8.6%)	16 (14%)	89 (19%)	
Ethnicity				0.006
Non-Hispanic	363 (95%)	112 (88%)	446 (89%)	
Hispanic	20 (5.2%)	15 (12%)	55 (11%)	
Primary insurance coverage†				<0.001
Government	224 (58%)	97 (75%)	413 (78%)	
Private insurance	155 (40%)	30 (23%)	111 (21%)	
Uninsured	4 (1.0%)	2 (1.6%)	3 (0.6%)	
Neighborhood-level index‡, median (IQR)				
ADI percentile	30 (20-43)	30 (20-48)	37 (24-52)	<0.001
SVI percentile	0.28 (0.13-0.52)	0.31 (0.13-0.54)	0.34 (0.19-0.66)	<0.001
Primary surgical service				0.011
Thoracic	137 (36%)	42 (32%)	221 (41%)	
Surgical oncology	93 (24%)	25 (19%)	86 (16%)	
Colorectal	153 (40%)	65 (49%)	227 (43%)	

Percentages might not add to 100% due to rounding. The Wilcoxon rank sum test was used for continuous variables. Chi-squared and Fisher's exact test were used for categorical variables, as appropriate. For respondents, demographic data were self-reported. For partial and non-respondents, demographics were abstracted from the electronic medical record (EHR). Partial and non-respondents with missing EHR demographic data were excluded from these analyses, which explains why the sum for each characteristic may be less than the total partial/non-respondent sample size. *Partial respondents completed a subset of the screener and were excluded from the study's final cohort. Non-respondents include those who declined to participate, those who were unable to be reached after three attempts, and those who initially consented but could not be reached at their scheduled survey time. †The definition of primary insurance coverage is outlined in the Supplemental Methods S1. Insurance data were abstracted from the EHR for all groups. ‡The national deprivation percentile was used for both neighborhood-level indices. Patients with non-numerical ADI ranks were excluded to maintain interpretability and comparability of deprivation percentiles. Abbreviations: IQR, interquartile range; ADI, Area Deprivation Index; SVI, Social Vulnerability Index.

Supplemental Table S2. Psychological and social vulnerabilities reported by patients undergoing major elective cancer surgery, stratified by primary surgical service.

**Psychosocial Domains, n (%)**	Colorectal Surgery (n=153)	Thoracic Surgery (n = 137)	Surgical Oncology (n=93)	p-value	q-value*
Psychological domains					
≥ Moderate anxiety	26 (17)	21 (15)	13 (14)	0.812	0.928
≥ Moderate depression	29 (19)	19 (14)	19 (20)	0.363	0.753
Lack of spirituality/religion	85 (56)	73 (53)	48 (52)	0.826	0.928
Low resilience	15 (9.8)	10 (7.3)	6 (6.5)	0.590	0.885
Limited resourcefulness	5 (3.3)	3 (2.2)	1 (1.1)	0.578	0.885
Anger	7 (4.6)	4 (2.9)	7 (7.5)	0.268	0.670
High-risk alcohol use	51 (33)	33 (24)	20 (22)	0.078	0.526
History of tobacco use	63 (41)	97 (71)	38 (41)	<0.001	<0.001
Current marijuana use	27 (18)	20 (15)	20 (22)	0.399	0.770
History of SUD	16 (10)	16 (12)	5 (5.4)	0.258	0.670
Social domains					
Food insecurity	10 (6.5)	7 (5.1)	5 (5.4)	0.860	0.928
Transportation needs	9 (5.9)	4 (2.9)	4 (4.3)	0.472	0.850
Housing insecurity	35 (23)	30 (22)	20 (22)	0.964	0.964
Utility difficulties	22 (14)	20 (15)	7 (7.5)	0.217	0.670
Intimate partner violence	4 (2.6)	1 (0.7)	1 (1.1)	0.581	0.885
Limited social support	33 (22)	24 (18)	8 (8.6)	0.031	0.419
Limited access to care	38 (25)	24 (18)	25 (27)	0.181	0.670
Low patient activation	5 (3.3)	3 (2.2)	2 (2.2)	0.849	0.928
Limited health literacy	12 (7.8)	11 (8.0)	5 (5.4)	0.711	0.914
High perceived stress	10 (6.5)	8 (5.8)	5 (5.4)	0.929	0.964
Limited community involvement	24 (16)	26 (19)	14 (15)	0.669	0.903
Limited surgeon trust	48 (31)	55 (40)	24 (26)	0.064	0.526
Everyday discrimination	4 (2.6)	9 (6.6)	4 (4.3)	0.263	0.670
Lack of access to healthy foods	41 (27)	41 (30)	19 (20)	0.273	0.670
Limited neighborhood recreation infrastructure	101 (66)	78 (57)	54 (58)	0.235	0.670
Lack of community cohesion and informal social control	113 (74)	96 (70)	73 (78)	0.362	0.753
Other HRSNs†	5 (3.3)	2 (1.5)	2 (2.2)	0.637	0.903

Domains, items, instruments, and scoring are listed in Supplemental Methods S2; binary operationalization is described in Supplemental Methods S3. *To account for multiple comparisons across the 27 psychosocial domains, a Benjamini-Hochberg correction was applied to control the false discovery rate while maintaining statistical power. Reported q-values reflect the adjusted significance levels. †Other HRSNs included clothing, childcare, and other self-reported social needs within the past 12 months. Abbreviations: HRSNs, health-related social needs; SUD, substance use disorder.

Supplemental Table S3. Post-hoc pairwise comparisons of psychological and social vulnerabilities by primary surgery service.

Psychosocial Domains	Statistical Test	Surgery Service 1	Surgery Service 2	p-value	q-value*
History of tobacco use	Pairwise Chi-square	Colorectal	Thoracic	<0.001	<0.001
History of tobacco use	Pairwise Chi-square	Colorectal	Surgical Oncology	1.00	1.00
History of tobacco use	Pairwise Chi-square	Thoracic	Surgical Oncology	<0.001	<0.001

Post hoc pairwise analyses were conducted using pairwise Chi-squared or Fisher's exact tests with Benjamini-Hochberg (BH) correction following a significant overall BH-adjusted test. This correction was applied to adjust for multiple comparisons across the three surgery services. *Reported q-values reflect the adjusted significance levels. Abbreviations: BH, Benjamini-Hochberg.

Supplemental Table S4. Psychological and social vulnerabilities reported by patients undergoing major elective cancer surgery, stratified by Area Deprivation Index.

**Psychosocial Domains, n (%)**	Low Area Deprivation Index (n=278)	High Area Deprivation Index (n=105)	p-value	q-value*
Psychological domains				
≥ Moderate anxiety	44 (16)	16 (15)	>0.999	>0.999
≥ Moderate depression	44 (16)	23 (22)	0.213	0.565
Lack of spirituality/religion	160 (58)	46 (44)	0.022	0.148
Low resilience	20 (7.2)	11 (10)	0.401	0.569
Limited resourcefulness	6 (2.2)	3 (2.9)	0.710	0.831
Anger	10 (3.6)	8 (7.6)	0.108	0.469
High-risk alcohol use	82 (29)	22 (21)	0.122	0.469
History of tobacco use	139 (50)	59 (56)	0.334	0.567
Current marijuana use	44 (16)	23 (22)	0.213	0.565
History of SUD	24 (8.6)	13 (12)	0.361	0.567
Social domains				
Food insecurity	7 (2.5)	15 (14)	<0.001	<0.001
Transportation needs	9 (3.2)	8 (7.6)	0.091	0.469
Housing insecurity	58 (21)	27 (26)	0.378	0.567
Utility difficulties	32 (12)	17 (16)	0.293	0.565
Intimate partner violence	3 (1.1)	3 (2.9)	0.352	0.567
Limited social support	45 (16)	20 (19)	0.608	0.782
Limited access to care	58 (21)	29 (28)	0.204	0.565
Low patient activation	8 (2.9)	2 (1.9)	0.734	0.831
Limited health literacy	14 (5.0)	14 (13)	0.010	0.093
High perceived stress	14 (5.0)	9 (8.6)	0.290	0.565
Limited community involvement	45 (16)	19 (18)	0.770	0.831
Limited surgeon trust	94 (34)	33 (31)	0.749	0.831
Everyday discrimination	10 (3.6)	7 (6.7)	0.263	0.565
Lack of access to healthy foods	76 (27)	25 (24)	0.569	0.769
Limited neighborhood recreation infrastructure	183 (66)	50 (48)	0.002	0.023
Lack of community cohesion and informal social control	200 (72)	82 (78)	0.276	0.565
Other HRSNs†	7 (2.5)	2 (1.9)	>0.999	>0.999

Patient's residential addresses were geocoded to the census block group level to determine their neighborhood's national ADI percentile. High deprivation was defined as ≥ 75th percentile. Patients with non-numeric ADI values were excluded to maintain interpretability and comparability of deprivation percentiles. Domains, items, instruments, and scoring are listed in Supplemental Methods S2; binary operationalization is described in Supplemental Methods S3. *To account for multiple comparisons across the 27 psychosocial domains, a Benjamini-Hochberg correction was applied to control the false discovery rate while maintaining statistical power. Reported q-values reflect the adjusted significance levels. †Other HRSNs included clothing, childcare, and other self-reported social needs within the past 12 months. Abbreviations: HRSNs, health-related social needs; SUD, substance use disorder.

Supplemental Table S5. Psychological and social vulnerabilities reported by patients undergoing major elective cancer surgery, stratified by self-identified gender.

**Psychosocial Domains, n (%)**	Self-Identified Woman (n=193)	Self-Identified Man (n=190)	p-value	q-value*
Psychological domains				
≥ Moderate anxiety	39 (20)	21 (11)	0.020	0.136
≥ Moderate depression	42 (22)	25 (13)	0.037	0.202
Lack of spirituality/religion	83 (43)	123 (65)	<0.001	<0.001
Low resilience	19 (9.8)	12 (6.3)	0.281	0.520
Limited resourcefulness	2 (1.0)	7 (3.7)	0.103	0.280
Anger	12 (6.2)	6 (3.2)	0.241	0.520
High-risk alcohol use	49 (25)	55 (29)	0.504	0.666
History of tobacco use	94 (49)	104 (55)	0.281	0.520
Current marijuana use	27 (14)	40 (21)	0.092	0.280
History of SUD	11 (5.7)	26 (14)	0.013	0.121
Social domains				
Food insecurity	11 (5.7)	11 (5.8)	>0.999	>0.999
Transportation needs	6 (3.1)	11 (5.8)	0.305	0.520
Housing insecurity	44 (23)	41 (22)	0.870	>0.999
Utility difficulties	20 (10)	29 (15)	0.200	0.490
Intimate partner violence	3 (1.6)	3 (1.6)	>0.999	>0.999
Limited social support	37 (19)	28 (15)	0.308	0.520
Limited access to care	40 (21)	47 (25)	0.415	0.590
Low patient activation	2 (1.0)	8 (4.2)	0.060	0.271
Limited health literacy	9 (4.7)	19 (10)	0.070	0.271
High perceived stress	14 (7.3)	9 (4.7)	0.411	0.590
Limited community involvement	21 (11)	43 (23)	0.003	0.044
Limited surgeon trust	56 (29)	71 (37)	0.104	0.280
Everyday discrimination	9 (4.7)	8 (4.2)	>0.999	>0.999
Lack of access to healthy foods	48 (25)	53 (28)	0.578	0.710
Limited neighborhood recreation infrastructure	121 (63)	112 (59)	0.518	0.666
Lack of community cohesion and informal social control	143 (74)	139 (73)	0.927	>0.999
Other HRSNs†	3 (1.6)	6 (3.2)	0.335	0.532

Domains, items, instruments, and scoring are listed in Supplemental Methods S2; binary operationalization is described in Supplemental Methods S3. *To account for multiple comparisons across the 27 psychosocial domains, a Benjamini-Hochberg correction was applied to control the false discovery rate while maintaining statistical power. Reported q-values reflect the adjusted significance levels. †Other HRSNs included clothing, childcare, and other self-reported social needs within the past 12 months. Abbreviations: HRSNs, health-related social needs; SUD, substance use disorder.

Supplemental Table S6. Psychological and social vulnerabilities reported by patients undergoing major elective cancer surgery, stratified by self-identified race and ethnicity.

**Psychosocial Domains, n (%)**	Self-Identified Non-Hispanic White (n=336)	Self-Identified Hispanic and/or Non-White (n=47)	p-value	q-value*
Psychological domains				
≥ Moderate anxiety	51 (15)	9 (19)	0.626	0.676
≥ Moderate depression	52 (15)	15 (32)	0.010	0.058
Lack of spirituality/religion	187 (56)	19 (40)	0.071	0.174
Low resilience	25 (7.4)	6 (13)	0.247	0.376
Limited resourcefulness	5 (1.5)	4 (8.5)	0.016	0.071
Anger	14 (4.2)	4 (8.5)	0.256	0.376
High-risk alcohol use	97 (29)	7 (15)	0.065	0.174
History of tobacco use	176 (52)	22 (47)	0.575	0.647
Current marijuana use	60 (18)	7 (15)	0.767	0.793
History of SUD	32 (9.5)	5 (11)	0.793	0.793
Social domains				
Food insecurity	12 (3.6)	10 (21)	<0.001	0.002
Transportation needs	13 (3.9)	4 (8.5)	0.142	0.296
Housing insecurity	72 (21)	13 (28)	0.438	0.514
Utility difficulties	37 (11)	12 (26)	0.011	0.058
Intimate partner violence	4 (1.2)	2 (4.3)	0.160	0.309
Limited social support	54 (16)	11 (23)	0.295	0.376
Limited access to care	73 (22)	14 (30)	0.294	0.376
Low patient activation	6 (1.8)	4 (8.5)	0.024	0.093
Limited health literacy	21 (6.3)	7 (15)	0.064	0.174
High perceived stress	18 (5.4)	5 (11)	0.181	0.327
Limited community involvement	51 (15)	13 (28)	0.052	0.174
Limited surgeon trust	106 (32)	21 (45)	0.104	0.234
Everyday discrimination	11 (3.3)	6 (13)	0.011	0.058
Lack of access to healthy foods	92 (27)	9 (19)	0.306	0.376
Limited neighborhood recreation infrastructure	216 (64)	17 (36)	<0.001	0.005
Lack of community cohesion and informal social control	244 (73)	38 (81)	0.306	0.376
Other HRSNs†	7 (2.1)	2 (4.3)	0.305	0.376

Domains, items, instruments, and scoring are listed in Supplemental Methods S2; binary operationalization is described in Supplemental Methods S3. *To account for multiple comparisons across the 27 psychosocial domains, a Benjamini-Hochberg correction was applied to control the false discovery rate while maintaining statistical power. Reported q-values reflect the adjusted significance levels. †Other HRSNs included clothing, childcare, and other self-reported social needs within the past 12 months. Abbreviations: HRSNs, health-related social needs; SUD, substance use disorder.

Supplemental Table S7. Post-hoc pairwise comparisons of psychological and social vulnerabilities by household income.

Psychosocial Domains	Statistical Test	Income Group 1	Income Group 2	p-value	q-value*
Anger	Pairwise Fisher	Below poverty	High income	0.079	0.119
Anger	Pairwise Fisher	Below poverty	Low income	0.066	0.119
Anger	Pairwise Fisher	Below poverty	Unknown	0.694	0.694
Anger	Pairwise Fisher	High income	Low income	0.608	0.694
Anger	Pairwise Fisher	High income	Unknown	0.001	0.003
Anger	Pairwise Fisher	Low income	Unknown	0.004	0.011
Limited resourcefulness	Pairwise Fisher	Below poverty	High income	0.002	0.01
Limited resourcefulness	Pairwise Fisher	Below poverty	Low income	0.016	0.047
Limited resourcefulness	Pairwise Fisher	Below poverty	Unknown	0.31	0.372
Limited resourcefulness	Pairwise Fisher	High income	Low income	1.00	1.00
Limited resourcefulness	Pairwise Fisher	High income	Unknown	0.072	0.144
Limited resourcefulness	Pairwise Fisher	Low income	Unknown	0.172	0.258
History of SUD	Pairwise Fisher	Below poverty	High income	0.019	0.092
History of SUD	Pairwise Fisher	Below poverty	Low income	0.451	0.541
History of SUD	Pairwise Fisher	Below poverty	Unknown	0.153	0.305
History of SUD	Pairwise Fisher	High income	Low income	0.031	0.092
History of SUD	Pairwise Fisher	High income	Unknown	1.00	1.00
History of SUD	Pairwise Fisher	Low income	Unknown	0.282	0.422
Food insecurity	Pairwise Fisher	Below poverty	High income	0.009	0.019
Food insecurity	Pairwise Fisher	Below poverty	Low income	0.422	0.634
Food insecurity	Pairwise Fisher	Below poverty	Unknown	1.00	1.00
Food insecurity	Pairwise Fisher	High income	Low income	0.009	0.019
Food insecurity	Pairwise Fisher	High income	Unknown	0.002	0.014
Food insecurity	Pairwise Fisher	Low income	Unknown	0.729	0.875
Transportation needs	Pairwise Fisher	Below poverty	High income	0.068	0.203
Transportation needs	Pairwise Fisher	Below poverty	Low income	0.284	0.387
Transportation needs	Pairwise Fisher	Below poverty	Unknown	1.00	1.00
Transportation needs	Pairwise Fisher	High income	Low income	0.323	0.387
Transportation needs	Pairwise Fisher	High income	Unknown	0.013	0.077
Transportation needs	Pairwise Fisher	Low income	Unknown	0.227	0.387
Utility difficulties	Pairwise Fisher	Below poverty	High income	0.007	0.029
Utility difficulties	Pairwise Fisher	Below poverty	Low income	0.476	0.572
Utility difficulties	Pairwise Fisher	Below poverty	Unknown	0.453	0.572
Utility difficulties	Pairwise Fisher	High income	Low income	0.01	0.029
Utility difficulties	Pairwise Fisher	High income	Unknown	0.041	0.083
Utility difficulties	Pairwise Chi-square	Low income	Unknown	1.00	1.00
Intimate partner violence	Pairwise Fisher	Below poverty	High income	0.079	0.159
Intimate partner violence	Pairwise Fisher	Below poverty	Low income	0.265	0.398
Intimate partner violence	Pairwise Fisher	Below poverty	Unknown	0.648	0.777
Intimate partner violence	Pairwise Fisher	High income	Low income	1.00	1.00
Intimate partner violence	Pairwise Fisher	High income	Unknown	<0.001	0.001
Intimate partner violence	Pairwise Fisher	Low income	Unknown	0.027	0.08
Limited social support	Pairwise Fisher	Below poverty	High income	0.031	0.122
Limited social support	Pairwise Fisher	Below poverty	Low income	0.476	0.715
Limited social support	Pairwise Fisher	Below poverty	Unknown	0.725	0.831
Limited social support	Pairwise Chi-square	High income	Low income	0.133	0.265
Limited social support	Pairwise Fisher	High income	Unknown	0.041	0.122
Limited social support	Pairwise Chi-square	Low income	Unknown	0.831	0.831
Limited access to care	Pairwise Fisher	Below poverty	High income	1.00	1.00

Limited access to care	Pairwise Fisher	Below poverty	Low income	0.703	0.844
Limited access to care	Pairwise Chi-square	Below poverty	Unknown	0.081	0.162
Limited access to care	Pairwise Chi-square	High income	Low income	0.676	0.844
Limited access to care	Pairwise Chi-square	High income	Unknown	0.001	0.008
Limited access to care	Pairwise Chi-square	Low income	Unknown	0.078	0.162
Low patient activation	Pairwise Fisher	Below poverty	High income	0.003	0.016
Low patient activation	Pairwise Fisher	Below poverty	Low income	0.016	0.047
Low patient activation	Pairwise Fisher	Below poverty	Unknown	0.31	0.372
Low patient activation	Pairwise Fisher	High income	Low income	1.00	1.00
Low patient activation	Pairwise Fisher	High income	Unknown	0.096	0.192
Low patient activation	Pairwise Fisher	Low income	Unknown	0.172	0.258
Limited health literacy	Pairwise Fisher	Below poverty	High income	0.025	0.076
Limited health literacy	Pairwise Fisher	Below poverty	Low income	1.00	1.00
Limited health literacy	Pairwise Fisher	Below poverty	Unknown	0.666	0.898
Limited health literacy	Pairwise Fisher	High income	Low income	0.003	0.018
Limited health literacy	Pairwise Fisher	High income	Unknown	0.042	0.083
Limited health literacy	Pairwise Fisher	Low income	Unknown	0.748	0.898
Other HRSNs*	Pairwise Fisher	Below poverty	High income	0.014	0.085
Other HRSNs	Pairwise Fisher	Below poverty	Low income	0.284	0.426
Other HRSNs	Pairwise Fisher	Below poverty	Unknown	0.589	0.707
Other HRSNs	Pairwise Fisher	High income	Low income	0.087	0.174
Other HRSNs	Pairwise Fisher	High income	Unknown	0.05	0.151
Other HRSNs	Pairwise Fisher	Low income	Unknown	1.00	1.00

Post hoc pairwise analyses were conducted using pairwise Chi-squared or Fisher's exact tests with Benjamini-Hochberg (BH) correction following a significant overall BH-adjusted test. This correction was applied to adjust for multiple comparisons across the four income groups. *Reported q-values reflect the adjusted significance levels. Income was categorized based on the U.S. Federal Poverty Line (FPL), adjusted for household size, number of children under 18, and the age of the householder. Low income was defined as an annual income below the U.S. FPL, middle income as between 100% and 200% of the FPL, and high income as above 200%. Patients who did not know their annual household income or declined to answer were categorized as "unknown." *Other HRSNs included clothing, childcare, and other self-reported social needs within the past 12 months. Abbreviations: BH, Benjamini-Hochberg; SUD, substance use disorder; HRSNs, health-related social needs; FPL, federal poverty line.
